# Follow-up interviews from The Salford Lung Study (COPD) and analyses per treatment and exacerbations

**DOI:** 10.1038/s41533-019-0123-0

**Published:** 2019-05-09

**Authors:** Diane Whalley, Henrik Svedsater, Lynda Doward, Rebecca Crawford, David Leather, James Lay-Flurrie, Nick Bosanquet

**Affiliations:** 1 RTI Health Solutions, Manchester, UK; 2 Value Evidence & Outcomes, GlaxoSmithKline plc., Brentford, Middlesex UK; 3 Global Respiratory Franchise, GlaxoSmithKline plc., Uxbridge, Middlesex UK; 4 Clinical Statistics, GlaxoSmithKline plc., Uxbridge, Middlesex UK; 5 Imperial College London, London, UK

**Keywords:** Quality of life, Respiratory signs and symptoms

## Abstract

The Salford Lung Study in chronic obstructive pulmonary disease (SLS COPD) was a 12-month, Phase III, open-label, randomised study comparing the effectiveness and safety of initiating once-daily fluticasone furoate 100 µg/vilanterol 25 µg (FF/VI) with continuing usual care (UC). Follow-up interviews were conducted among a subset of 400 patients who completed SLS COPD to further understand patients’ experiences with treatment outcomes and the impact of COPD, and potential risk factors associated with higher rates of exacerbations during SLS COPD. Another objective was to explore how such patient-centred outcomes differed by randomised treatment. Patients’ perceived control over COPD and effects on quality of life (QoL) were similar between treatment groups at the time of the follow-up interview, but more patients in the FF/VI group compared with UC reported perceived improvements in COPD control and QoL during the study. Of patients who experienced ≥2 exacerbations during SLS COPD, a greater percentage were women, were unemployed or homemakers, or were on long-term sick leave. Having ≥2 exacerbations also appeared to be associated with smoking, seeing a hospital specialist, a feeling of having no/little control over COPD, perceived worsening of feelings of control and reduced overall QoL since the start of the study, being aware of impending exacerbation occurrence and a more severe last exacerbation. Initiation of FF/VI was associated with a greater perceived improvement in patients’ control of their COPD and QoL throughout SLS COPD than continuation of UC. Suggestions that smoking status and feelings of control are potentially related to exacerbation require further investigation.

## Chronic lung disease: examining patient perceptions after trial

The Salford Lung Study was a 12-month trial during which patients were either given the drug fluticasone furoate/vilanterol (FF/VI) in addition to existing treatment or continued their usual medication. Henrik Svedsater at GlaxoSmithKline plc. UK and co-workers conducted follow-up interviews with 400 study participants to examine patient perceptions of trial outcomes, COPD management and disease treatments. Throughout the drug trial, patients given FF/VI were convinced that their control of COPD and quality of life improved. During the follow-up interviews both groups had similar perceptions of these two elements, but perceived improvement in COPD control was generally greater with FF/VI. Many participants felt that suffering multiple exacerbations was linked primarily to smoking and perceived worsening of control; this warrants further investigation.

## Introduction

Chronic obstructive pulmonary disease (COPD) is one of the most common respiratory conditions in the UK, characterised by chronic airflow limitation and persistent respiratory symptoms including breathlessness (dyspnoea), cough and sputum production.^1^ COPD is associated with substantial economic and social burden, with symptoms impacting on every aspect of a person’s day-to-day life.^1,2^ Much of this burden is driven by acute exacerbations of respiratory symptoms, which occur with variable frequency and result in increased medication use, visits to healthcare practitioners and hospitalisation.^1^ As well as the physical burden of living with symptoms of the disease, COPD has a significant psychological burden, not only on patients,^3–6^ but also on their caregivers.^7,8^

Despite awareness among clinicians that the symptoms of COPD affect many areas of patients’ lives, the impact these symptoms have on individuals’ physical abilities are often underestimated.^5,6^ Clinicians focus on prevention of lung-function decline and ability to control symptoms as important when making treatment decisions, whereas for patients, treatment benefits include factors such as a desire to maintain their lifestyle.^6^ This is crucial as, for example, patient-defined benefits can play a role in treatment adherence; non-adherence has been linked with feelings of wanting to be in control and not having to depend on treatment, rather than just a desire to control symptoms.^6^ Thus, it is important to evaluate patient perceptions of their disease and its impact on their lives.^6^

Clinical trials of new therapies in COPD often impose rigid eligibility criteria, limiting extrapolation of treatment effects to the wider population since selected patients are potentially not representative of those seen in routine clinical practice.^9–11^ For example, patients in real-world settings tend to be older and have more comorbidities and worse quality of life (QoL) scores than those traditionally included in clinical trials.^12,13^ Studies have shown that up to 83% of people treated for COPD in clinical practice would be ineligible to participate in randomised controlled trials.^10,13–17^ Furthermore, the perceived impact of COPD is infrequently evaluated, and QoL assessments are not typically prioritised in clinical trial reporting.

The Salford Lung Study in COPD (SLS COPD; NCT01551758) was a 12-month, Phase III, open-label, randomised, controlled clinical trial that compared the effectiveness and safety of initiating treatment with the once-daily inhaled corticosteroid (ICS)/long-acting beta_2_-agonist (LABA) combination of fluticasone furoate (FF) 100 µg/vilanterol (VI) 25 µg with continuing usual COPD maintenance therapy.^18^ The study was conducted in a large population of patients with COPD in conditions of routine clinical care at more than 80 general practitioner sites and 130 community pharmacies who had no previous experience in clinical trials. Unique features of the study included the writing of standard operating procedures by a local pharmacy steering group, the conduct of good clinical practice training for >2000 primary care providers and the development of IT software to extract data from NHS systems in near real time. Results showed that the initiation of once-daily treatment with FF/VI was associated with a lower rate of exacerbations than continuing usual care, including ICS/LABA combinations, without an increased risk of serious adverse events.^18^

As part of the SLS COPD study, follow-up interviews were conducted in a subset of 400 patients completing the trial to explore patient-centred outcomes not captured within the main SLS COPD, and to further understand the impact of COPD on patients’ lives. The experiences of all SLS COPD follow-up interview participants together have been reported previously.^19^ These showed that breathlessness was the dominant symptom for patients in SLS COPD, and that this symptom had the greatest impact on participants’ daily functioning, inhibiting their ability to perform physical activities.^19^

Here we report further analyses of the follow-up interview data according to randomised treatment group, as well as an evaluation of factors potentially associated with COPD exacerbations.

## Results

### Participants

**Fig. 1:**
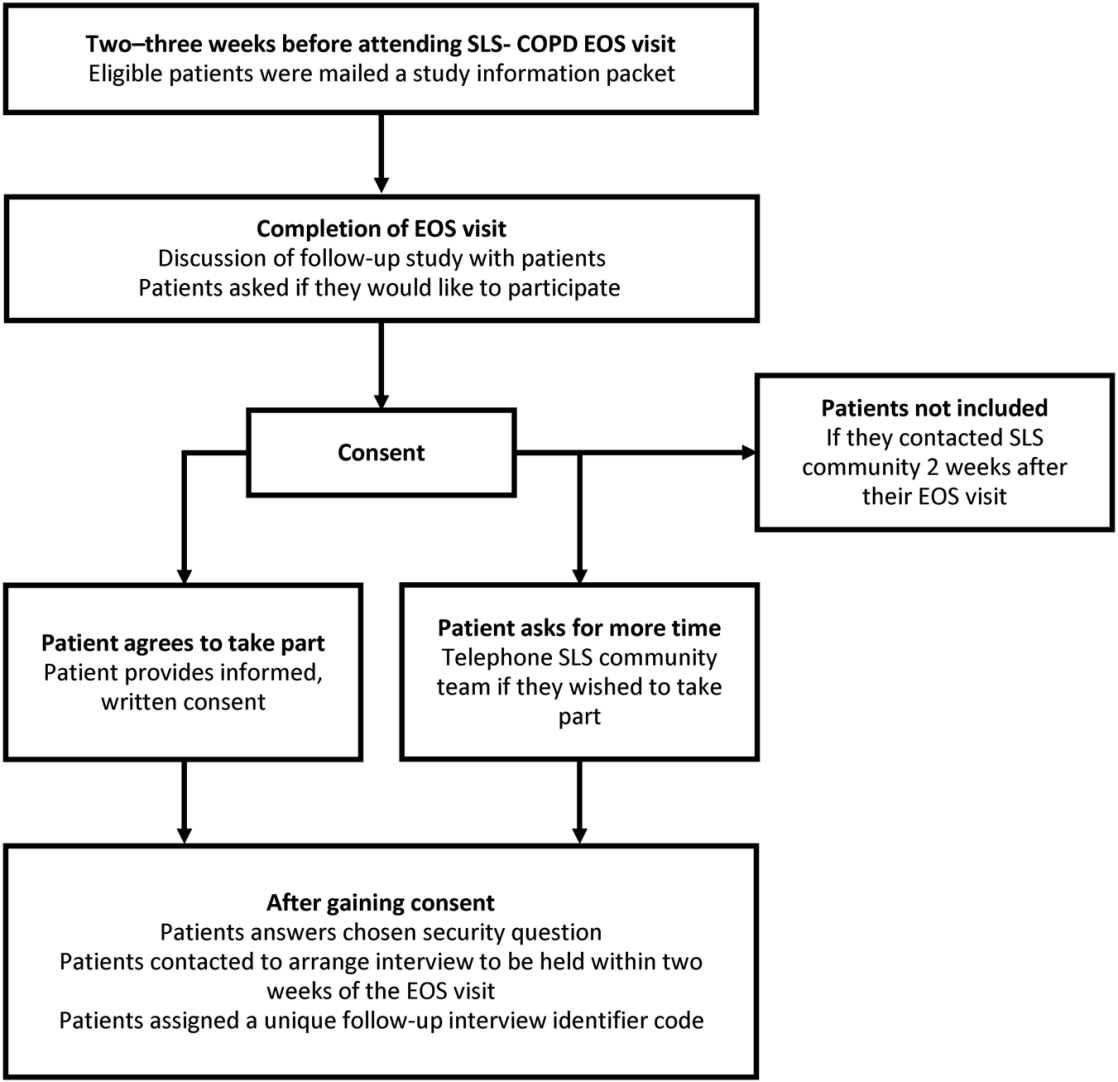
Patient recruitment and consent process *COPD* chronic obstructive pulmonary disease, *EOS* end-of-study, *SLS COPD* The Salford Lung Study in COPD

The process of patient recruitment and consent for the follow-up interviews is shown in Fig. 1. The 400 patients who participated in the follow-up interviews had a mean age of 65.2 years at the time of randomisation in SLS COPD and 53.8% were male. Over half of the participants (56.3%) were cohabiting with a partner. Only 11.4% of participants were working in a full-time, part-time or voluntary capacity; 67.0% were retired, 12.3% were unemployed or homemakers and 5% were on long-term sick leave. More than one-third of participants (37%) were current smokers while 53.8% were former smokers. More than 60% of participants reported having at least one other long-term illness or health condition in addition to their COPD; 43.8% had an illness that limited their physical activity or mobility and 5.3% reported having a psychological or emotional condition.

In the year prior to SLS COPD, 20% of participants had experienced no exacerbations while 27% had experienced one exacerbation and 53% had experienced two or more exacerbations. During SLS COPD, 31.3, 23.3 and 45.5% of participants experienced none, one, or at least two exacerbations, respectively. Of those subjects who participated in the exit interviews, approximately half had been randomised to initiate treatment with FF/VI 100/25 μg (48.3%) while the other half continued usual care (51.8%). Characteristics of the follow-up participants according to number of COPD exacerbations they experienced during SLS COPD are shown in Supplementary Table 1.

The characteristics of the follow-up participants have been previously shown to be reasonably representative of the overall sample of patients who completed SLS COPD (*n* = 2600) in terms of age, gender and exacerbation history in the year prior to SLS COPD.^19^

### Differences between participants, according to initiated treatment group

#### Perceived control over COPD

**Fig. 2:**
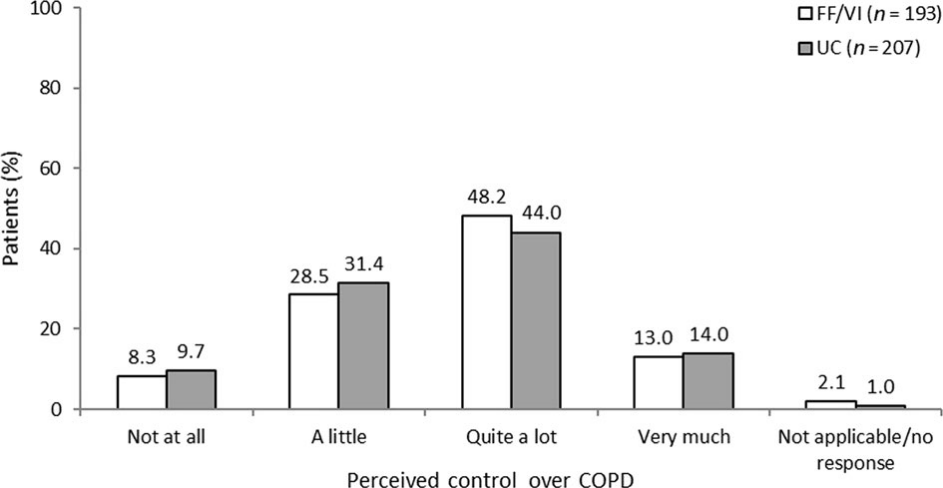
Perceived control over COPD during SLS COPD *FF/VI* participants initiated treatment with fluticasone furoate/vilanterol, *QoL* quality of life, *SLS COPD* Salford Lung Study in patients with chronic obstructive pulmonary disease, *UC* usual care

**Fig. 3:**
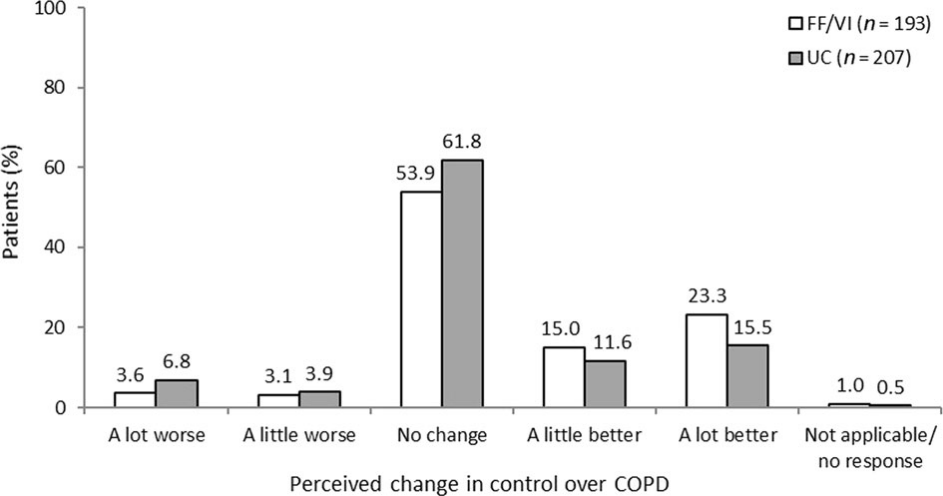
Perceived change in control over COPD during SLS COPD *FF/VI* participants initiated treatment with fluticasone furoate/vilanterol, *QoL* quality of life, *SLS COPD* Salford Lung Study in patients with chronic obstructive pulmonary disease, *UC* usual care

Participants’ perceptions of their control over COPD and change in perceived control during the course of SLS COPD are shown in Fig. 2 and Fig. 3, respectively. The reported perceived levels of control patients had over their COPD at the time of the follow-up interview were similar between the FF/VI and usual care treatment groups. However, more patients in the FF/VI group than in the usual care group reported an improvement in their perceived control over COPD during the study (38.3% [74/193] vs 27.1% [56/207], respectively). This was particularly noticeable in the percentage of participants who reported that their COPD had improved ‘a lot’ since the start of SLS COPD (23.3% [45/193] vs 15.5% [32/207], respectively).

#### Reported impact of COPD on participants’ QoL

The mean (standard deviation [SD]) overall QoL score (assessed on a response scale of 1–10, with higher scores indicating better QoL) was 6.5 (2.1) in both the FF/VI and usual care treatment groups. The impact of COPD on the QoL domains of functioning, activities, relationships, psychological well-being and independence assessed on a scale of 1–4, with higher scores indicating greater impact were similar in both treatment groups, with mean scores of 2.0–2.5 across all domains in the FF/VI group and 2.1–2.5 in the usual care group. The highest scores were recorded in the functioning and activities domains.

The impact of COPD on various aspects of participants’ daily lives within each of these QoL domains is shown in Table 1. Similar mean scores were observed for the individual questions on life area impact among patients randomised to initiate treatment with FF/VI or continue usual care. Climbing stairs or lifting/carrying were movements or activities within the functioning domain that were most impacted by COPD in both groups, with mean scores of 2.8–2.9 and 2.6–2.7, respectively. Within the activities domain, COPD had the greatest impact on physical activities (mean score of 2.7 in both treatment groups) and least impact on personal care (mean score of 1.5 in both treatment groups). Slight differences in mean scores between the FF/VI and usual care groups were observed for ‘loss of independence’ in the independence domain (1.6 [SD 1.0] vs 1.8; [SD 1.0]) and for ‘get anxious or worried about my COPD’ (1.8 [SD 1.0] vs 2.0 [SD 1.0]), and ‘find coughing embarrassing’ (2.0 [SD 1.1] vs 2.2 [SD 1.1]) in the psychological domain. In each of these cases, more patients randomised to usual care than to FF/VI reported greater (i.e., worse) impacts of COPD on these issues.

**Table 1:** Reported impact of COPD on QoL

Aspects of daily life impacted by COPD	Degree of COPD impact Mean score (SD), *n*				
	SLS COPD randomised treatment group		SLS COPD exacerbation rate		
	FF/VI (*n* = 193)	UC (*n* = 207)	0 (*n* = 125)	1 (*n* = 93)	≥2 (*n* = 182)
Functioning					
Bending down^a^	2.0 (1.0); 187	2.1 (1.0); 200	1.8 (0.9); 121	1.9 (1.0); 90	2.2 (1.0); 176
Lifting or carrying^a^	2.6 (1.1); 170	2.7 (1.1); 179	2.4 (1.1); 109	2.3 (1.0); 85	3.0 (1.0); 155
Walking outside^a^	2.0 (1.1); 186	2.0 (1.0); 200	1.8 (0.9); 118	1.9 (1.0); 91	2.3 (1.1); 177
Climbing stairs^a^	2.8 (1.0); 185	2.9 (0.9); 197	2.6 (1.0); 122	2.8 (0.9); 89	3.1 (0.9); 171
Talking^a^	1.5 (0.7); 190	1.5 (0.8); 204	1.3 (0.5); 124	1.5 (0.8); 91	1.6 (0.8); 179
Activities					
Physical activities^a^	2.7 (1.0); 182	2.7 (1.1); 189	2.5 (1.1); 113	2.5 (1.1); 90	2.9 (1.0); 168
Household jobs^a^	2.2 (1.1); 179	2.3 (1.1); 197	2.0 (1.0); 116	2.1 (1.1); 91	2.4 (1.1); 169
Local shopping^a^	1.8 (1.1); 144	1.9 (1.1); 158	1.7 (1.0); 99	1.7 (0.9); 69	2.1 (1.2); 134
Main shopping^a^	2.2 (1.1); 134	2.3 (1.2); 132	2.0 (1.2); 82	2.2 (1.2); 72	2.4 (1.2); 112
Personal care^a^	1.5 (0.9); 192	1.5 (0.8); 204	1.4 (0.8); 124	1.4 (0.8); 93	1.6 (1.0); 179
Relationships					
Relationship with partner^a^	1.7 (0.9), 119	1.7 (1.0), 130	1.7 (0.9), 78	1.6 (0.9), 67	1.8 (1.0), 104
Helping/doing things with family^a^	2.3 (1.0), 182	2.3 (1.0), 193	2.1 (1.0), 117	2.2 (1.0), 86	2.5 (1.0), 172
Socialising^a^	1.9 (1.1), 168	1.9 (1.1), 169	1.7 (0.9), 106	1.7 (1.1), 81	2.1 (1.1), 150
Holidays or days out^a^	1.9 (1.1), 165	2.0 (1.1), 174	1.8 (1.0), 105	1.7 (1.0), 82	2.2 (1.1), 152
Psychological					
Find coughing embarrassing^a^	2.0 (1.1), 167	2.2 (1.1), 185	1.9 (1.1), 106	2.1 (1.0), 82	2.3 (1.1), 164
Get anxious/worried^a^	1.8 (1.0), 193	2.0 (1.0), 206	1.7 (0.9), 125	1.9 (1.0), 93	2.0 (1.1), 181
Feel burden on family^a^	1.6 (1.0), 191	1.6 (1.0), 200	1.5 (0.9), 122	1.4 (0.8), 89	1.8 (1.1), 180
Independence					
Forced to plan activities^a^	1.9 (1.1), 190	1.9 (1.1), 201	1.7 (1.0), 125	1.7 (1.0), 90	2.1 (1.1), 176
Lost independence^a^	1.6 (1.0), 193	1.8 (1.0), 206	1.6 (0.9), 124	1.5 (0.9), 93	1.8 (1.1), 182
Feel trapped in house^a^	1.6 (0.9), 192	1.6 (1.0), 207	1.5 (0.9), 125	1.5 (0.9), 92	1.7 (1.0), 182

*COPD* chronic obstructive pulmonary disease, *FF/VI* fluticasone furoate/vilanterol, *QoL* quality of life, *SD* standard deviation, *SLS COPD* Salford Lung Study in patients with chronic obstructive pulmonary disease, *UC* usual care

^a^Assessed as: 1, not at all; 2, a little; 3, quite a lot and 4, very much/unable to do

**Fig. 4:**
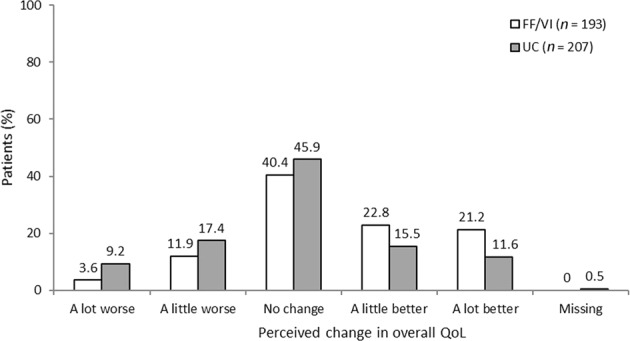
Perceived change in overall QoL during SLS COPD *FF/VI* participants initiated treatment with fluticasone furoate/vilanterol, *QoL* quality of life, *SLS COPD* Salford Lung Study in patients with chronic obstructive pulmonary disease, *UC* usual care

**Fig. 5:**
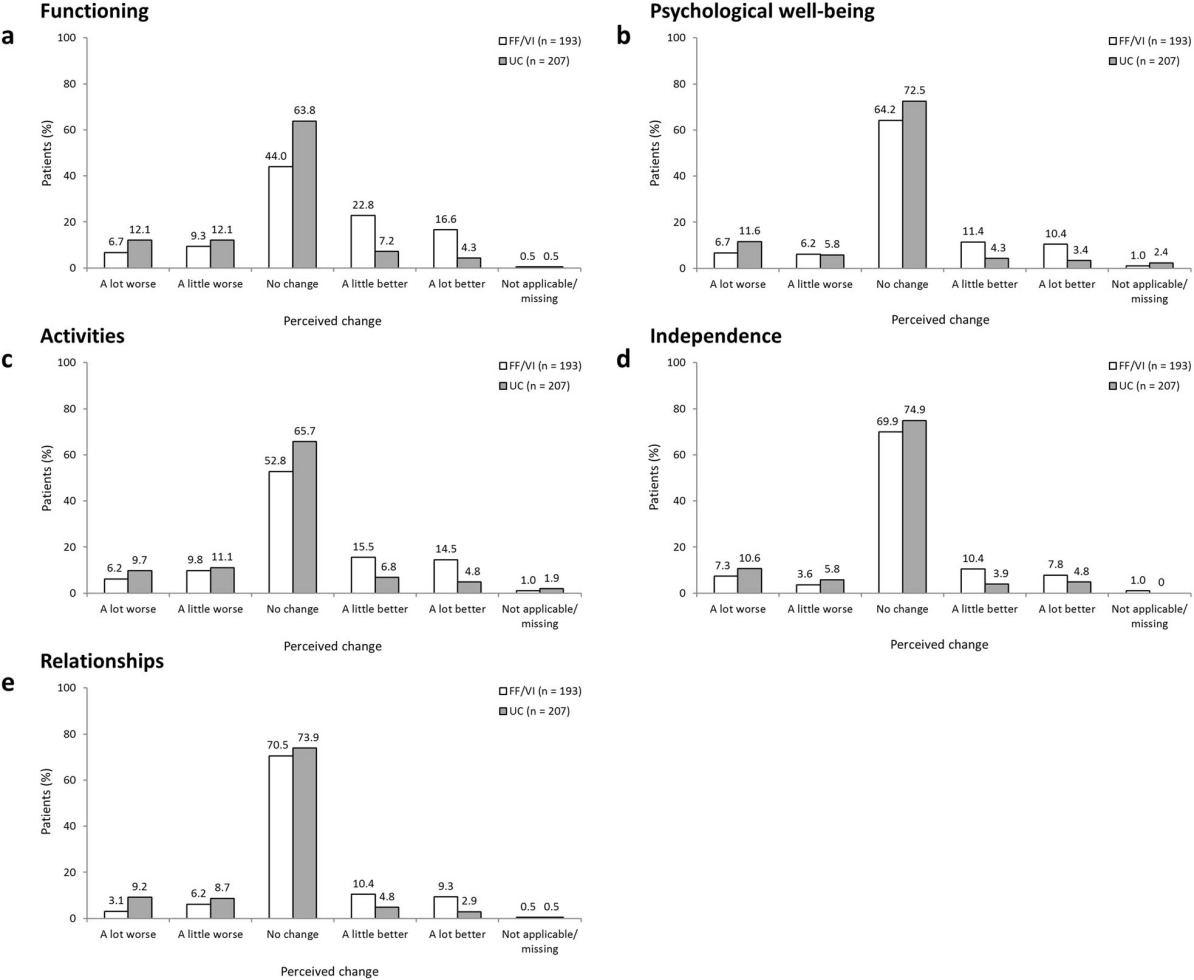
**a, b, c, d, e**. Perceived change in the daily life impact of COPD on QoL domains during SLS COPD *QoL* quality of life, *SLS COPD* Salford Lung Study in patients with chronic obstructive pulmonary disease, *UC* usual care

Perceived change in overall QoL and in the daily life impact of COPD on the QoL domains during SLS COPD are shown in Fig. 4 and Fig. 5a–e, respectively. A higher percentage of patients randomised to FF/VI than those randomised to usual care reported an improvement in overall QoL since the start of SLS COPD (44.4 vs 27.1%, respectively); more patients receiving usual care felt that their overall QoL had worsened compared with those randomised to initiate FF/VI (26.6 vs 15.5%, respectively; Fig. 4). A higher percentage of patients randomised to FF/VI than to usual care also reported an improvement in the effect of daily life impact across all QoL domains (Fig. 5a–e). These results were particularly evident for the functioning and activities domains, where the difference between treatment groups reporting an improvement was approximately 3-times higher among patients randomised to FF/VI versus usual care (functioning: 39.4 vs 11.5%; activities 30.0 vs 11.6%, respectively; Fig. 5a, c).

#### Reported sleep impairment

For the COPD and Asthma Sleep Impact Scale (CASIS), similar scores were observed across the two treatment groups. Mean (SD) scores were 43.7 (27.9) for FF/VI and 43.9 (28.9) for usual care. Median scores were slightly lower (less sleep impairment) for FF/VI than for usual care (42.9 and 46.4, respectively).

### Relationships between exacerbation rates and key patient-centred variables

#### Sociodemographic and lifestyle characteristics

In general, there was little association between the exacerbation rate during SLS COPD and many of the sociodemographic, health and disease factors explored in the follow-up interviews (Supplementary Tables 1, 2 and 3). Of the patients not experiencing exacerbations, 62.4% (78/125) were male and 37.6% (47/125) were female. This trend was then replicated among the patients experiencing one exacerbation, with 60.2% (56/93) being male and 39.8% (37/93) being female. For patients experiencing two or more exacerbations, the majority were female (56.6% female [103/182] vs 43.4% male [79/182]; Supplementary Table 1). Relationship status and employment status were broadly similar between patients with no, one, or two or more exacerbations (Supplementary Table 1). A higher percentage of patients who experienced two or more exacerbations (12.6% [23/182]) saw a hospital specialist for their COPD compared with those with no exacerbations (5.6% [7/125]) or one (5.4% [5/93]) exacerbation (Supplementary Table 2). The same was true of patients seeing respiratory nurses; 18.1% (33/182) of patients that experienced two or more exacerbations saw a respiratory nurse compared with 13.6% (17/125) that experienced none and 10.8% (10/93) that experienced one. Similar proportions of patients saw general practitioners and practice nurses for COPD (Supplementary Table 2). A higher percentage of patients who experienced two or more exacerbations were current smokers (40.7% [74/182]) compared with those having no exacerbations (32.8% [41/125]) or one exacerbation (35.5% [33/93]). Among patients who had ever experienced an exacerbation (*n* = 266), those who experienced two or more exacerbations in SLS COPD were the most frequent of the exacerbation subgroups to report that they would be aware when an exacerbation is about to happen (69.9% [102/146]) (Supplementary Table 3). For patients who experienced one exacerbation or no exacerbations in SLS COPD, 58.1% (36/62) and 53.4% (31/58), respectively, reported that they would be aware of an exacerbation about to happen.

#### Perceived control over COPD

**Fig. 6:**
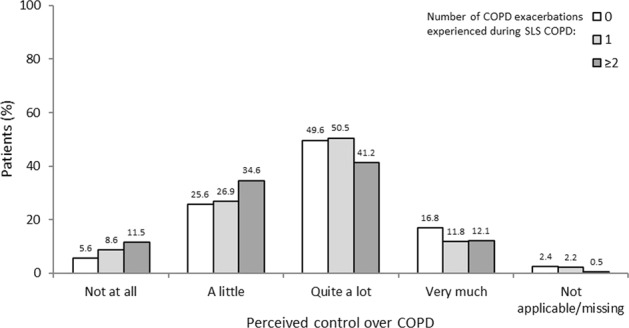
Perceived control over COPD according to the number of COPD exacerbations experienced during SLS COPD

**Fig. 7:**
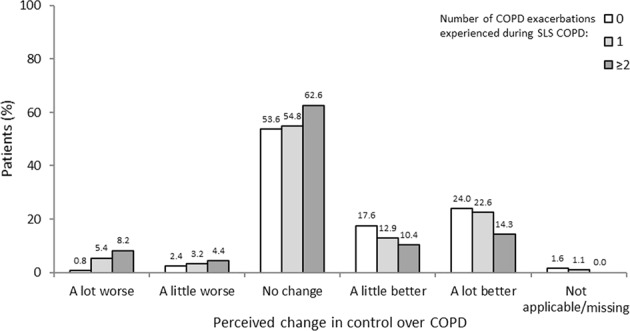
Perceived change in control over COPD according to the number of COPD exacerbations experienced during SLS COPD

A greater percentage of patients who had experienced two or more exacerbations during SLS COPD reported having ‘no control’ or ‘little control’ over their COPD compared with those who had experienced no exacerbations during the study (46.2 vs 31.2%, respectively; Fig. 6). Moreover, more patients who had experienced two or more exacerbations during SLS COPD reported that their feelings of control had worsened either ‘a lot’ or ‘a little’ since the start of SLS COPD compared with patients who experienced no exacerbations (12.6 vs 3.2%, respectively; Fig. 7). Conversely, fewer patients who experienced two or more exacerbations reported a perceived improvement in feelings of control over COPD since the start of SLS COPD compared with those who had one or no exacerbations (24.7 vs 35.5% and 41.6%, respectively; Fig. 7). Additional data are reported in Supplementary Table 3.

### Reported impact of COPD on participants’ QoL and coping mechanisms

Patients who experienced a greater number of exacerbations during SLS COPD reported a worse impact of COPD on multiple aspects of daily life (Table 1). Mean (SD) scores on the overall QoL questionnaire (assessed on a scale of 1−10, with higher scores indicating better QoL) were 6.9 (2.2) for patients with no exacerbations, 6.7 (2.0) for patients with one exacerbation, and 6.2 (2.0) for patients with two or more exacerbations during SLS COPD. Fewer patients who experienced two or more exacerbations reported an improvement in overall QoL since the start of SLS COPD compared with those with one or no exacerbations (26.4 vs 36.6% and 47.2%, respectively), and more felt that their overall QoL had worsened during the study (31.9, 17.2 and 8.8% for patients with two or more, one, or no exacerbations, respectively).

**Fig. 8:**
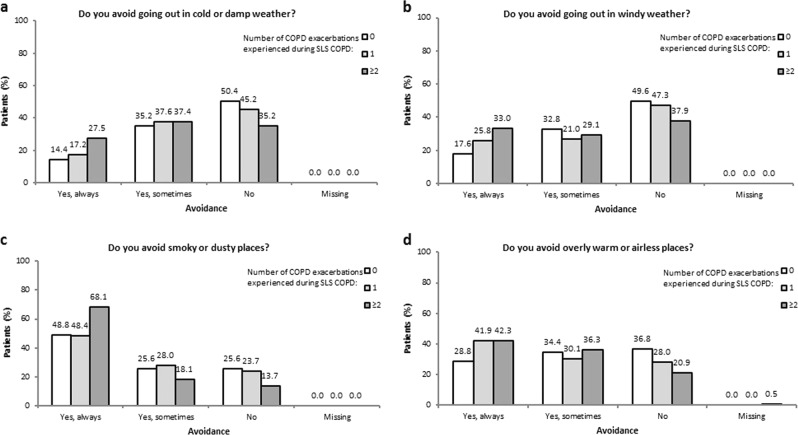
**a, b, c, d**. SLS COPD follow-up interview participants’ avoidance of perceived triggers for COPD exacerbations according to the number of COPD exacerbations experienced during SLS COPD

**Fig. 9:**
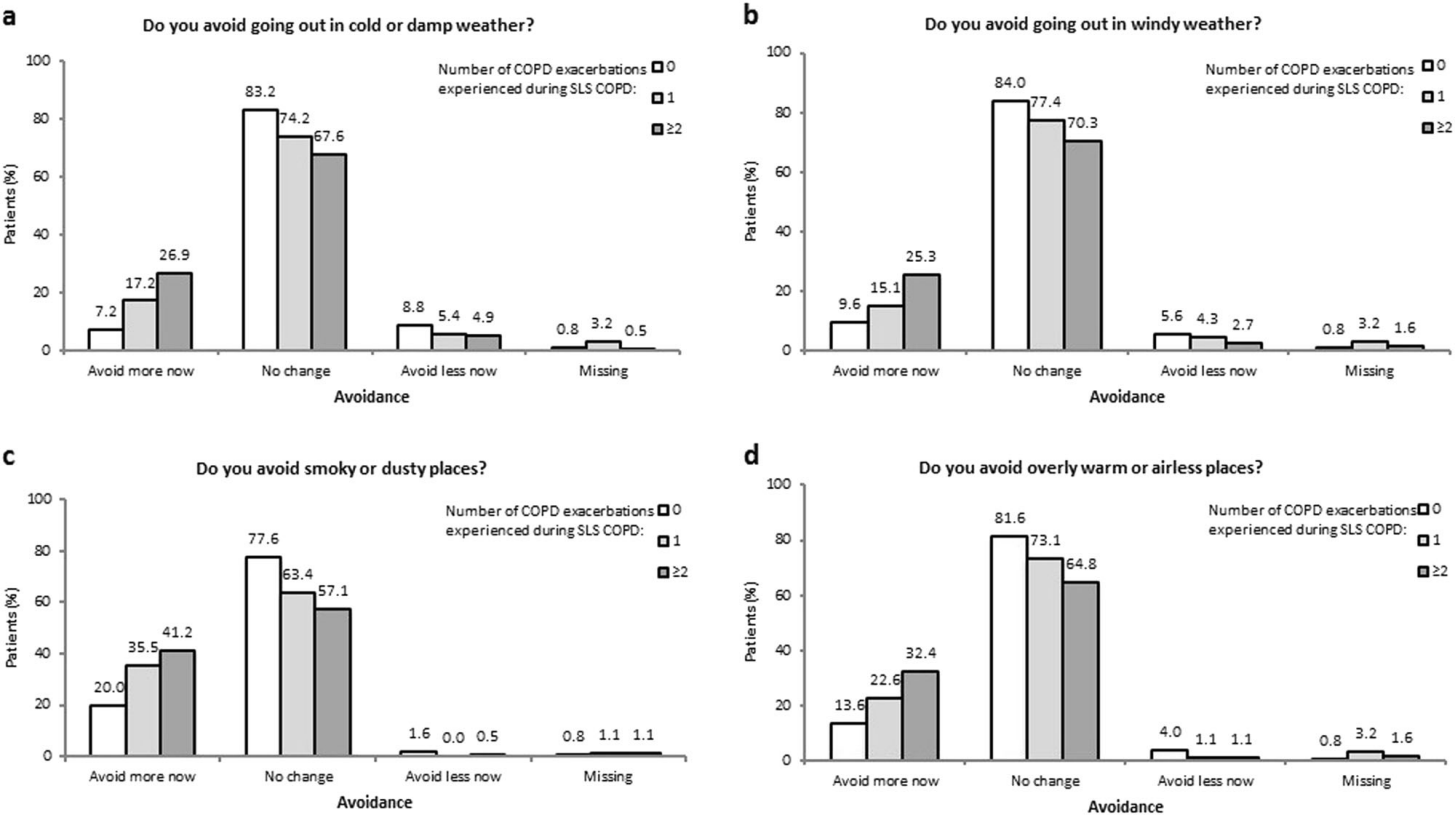
**a, b, c, d**. Change in SLS COPD follow-up interview participants’ avoidance of perceived triggers for COPD exacerbations according to the number of COPD exacerbations experienced during SLS COPD

With regard to perceived environmental triggers (Fig. 8a–d and Fig. 9a–d), the percentage of patients who ‘always’ avoid such triggers ranged from 27.5−68.1% for those who experienced two or more exacerbations during SLS COPD, compared with 17.2–48.4% and 14.4–48.8% for patients who experienced one or no exacerbations during SLS COPD, respectively (Fig. 8a–d). A greater percentage of patients with two or more exacerbations reported that they avoided these perceived triggers more since the start of SLS COPD compared with patients who experienced one or no exacerbations (Fig. 9a–d).

## Discussion

While patients’ perceptions of control over their COPD and perceived impacts of COPD on daily life and overall QoL were similar at the time of the follow-up interviews in patients who initiated FF/VI and those who continued usual care, there were clear differences in favour of the FF/VI treatment group on perceived changes in these issues since the start of SLS COPD. Specifically, a greater percentage of patients initiated on FF/VI perceived an improvement in control over COPD, and also reported improvement in QoL issues experienced in different domains of life and in overall QoL. While this could indicate bias (e.g., interviewer or response bias), other studies have also noted different outcomes between conventional longitudinal evaluation of change and patient-perceived change.^20^ This may be due to patient understanding of the subjective construct (e.g., QoL) under evaluation changing over time (response shift) or to recall bias by the patient. Such bias may have impacted patients randomised to FF/VI more than those randomised to continue usual care due to the novel treatment experience of patients in this group. The need for patients initiating FF/VI to learn new behaviours (such as inhaler technique on a new device and potentially new treatment effects) may have subsequently impacted their recollection of these behaviours and events. The fact that the results are consistent across different patient-centred outcomes suggests that it is less likely that this is simply an interviewer effect. Moreover, asking patients how various aspects of their COPD have improved or worsened since their last visit is typically how disease progression is captured in clinical practice, where doctors commonly ask and discuss with the patients how their condition has changed since the previous consultation and/or since a change of treatment was initiated.

Little association was observed between exacerbation rate and many of the sociodemographic, disease and health factors explored in the follow-up interviews. Males and those working full time represented the smallest percentages of patients with two or more exacerbations during SLS COPD. The analyses further suggested that having had two or more exacerbations during SLS COPD may be associated with generally seeing a hospital specialist in relation to COPD, currently smoking, the feeling of having no or little control over COPD, perceived worsening of feelings of control, reduced overall QoL since the start of SLS COPD, being aware of when an exacerbation was about to occur and having had a more severe last exacerbation. It should be noted, however, that the patient numbers in some of these groups are very small and therefore these results should be viewed with caution.

The results obtained here also demonstrate that, among patients who experienced two or more exacerbations, more reported a worsening in their QoL (31.8%) than reported an improvement (26.3%). This is to be expected, given that the negative correlation existing between QoL and COPD severity and exacerbations is widely accepted.^21^ Nevertheless, these outcomes also highlight the potential of the interventions studied in SLS COPD to make a meaningful difference to the lives of patients experiencing a number of exacerbations. It is also worth noting that the numeric differences in overall QoL scores between patients experiencing different numbers of exacerbations was lower than might be expected in the SLS COPD population; previous studies have highlighted associations between COPD exacerbations and health-related QoL.^22–24^

In the primary analysis of follow-up interview data from SLS COPD, COPD symptoms, breathlessness in particular, were found to have a significant impact on mobility and, in turn, QoL.^19^ The findings of the analyses reported here, of perceived improvements in feelings of disease control and improvement in QoL for patients who initiated treatment with FF/VI, are consistent with the findings for the entire SLS COPD population, who experienced a lower rate of exacerbations than patients who received usual care.^18^ Many factors may influence patients’ perceptions of disease control and QoL. For example, patients who experience fewer symptoms attributed to COPD, who have a better understanding of their disease, who experience better treatment control and who have less of an emotional response have been shown to have improved QoL and experience less impact of COPD on their daily life.^25^ In addition, it has been suggested that partnership-based management programmes may improve patient perceptions of the intrusiveness of their disease.^26^

The overall incidence of exacerbations in SLS COPD (66.5%)^19^ was much higher than has been reported in similar effectiveness studies such as the SubPopulations and InteRmediate Outcome Measures in COPD Study (SPIROMICS) (48.7%).^27^ While this high exacerbation rate limits the generalisability of SLS COPD results to populations with lower rates of exacerbation, it also provides an enhanced opportunity to study the relationship between patient and treatment factors with exacerbation rates within the SLS COPD cohort. Females have been reported as more likely to experience more frequent exacerbations than males,^28^ with the former having a 25% higher rate of exacerbations.^29^ This trend was reflected in SLS COPD. Conversely, in contrast to the findings of the current study regarding employment status, unemployed patients have previously been found to be at a lower risk of experiencing COPD exacerbations as measured by prednisolone use.^30^ The discrepancy in these findings may be a result of differences in the way COPD exacerbations and employment status were defined and measured across studies. For example, in SLS COPD, exacerbations were reported by patients through closed-ended questionnaires and were defined in the interview schedule as ‘an episode where your symptoms become much worse, and you need to change your treatment or you may need to seek medical help’. Exacerbation information in SPIROMICS was then gathered from patients prospectively by study investigators through structured telephone questionnaires and three annual clinical visits with patients under the definition ‘health care utilisation events (office visit, hospital admission, or Emergency Department visit for a respiratory “flare-up”) that involved the use of antibiotics and/or systemic corticosteroids’.^27^

The fact that follow-up interviews aimed to collect patient-centred data that are not typically captured within the context of a randomised controlled clinical trial is a particular strength of this study. The advantage of using patients who completed SLS COPD for this analysis was that it allowed the follow-up study data to be explored in relation to clinical outcomes and standardised patient-reported outcome assessments. Participants who completed the follow-up interviews were reasonably representative of the overall SLS COPD population; the subgroup analyses presented therefore provide further context for the findings from the main SLS COPD, and allows further exploration of patient-centred factors that might be associated with higher rates of exacerbation.

A limitation is that only patients who completed SLS COPD were eligible for participation in this follow-up study. Furthermore, not all patients approached for inclusion agreed to participate; therefore, the interview sample may not be representative of the overall SLS COPD population. Moreover, this was an exploratory study, and as such the data were analysed descriptively, with no statistical tests of group differences conducted. Therefore, no conclusions can be made regarding the significance or meaning of the numeric differences observed. When interpreting the findings from the follow-up interviews, it is important to view these in the context of the main SLS study and results.

This was an exploratory study designed to allow the consideration of a broad range of issues of potential relevance to outcomes in COPD. Owing to the exploratory nature of this work, meaning the study was not designed with the specific purpose of obtaining this information prospectively, limited inferences can be made from the results and definitive conclusions should not be drawn. In addition, caution should be taken when generalising results to the wider COPD population due to the apparent differences between the population included here, such as high exacerbation frequency, and usual COPD populations in clinical settings. Further studies are required on possible predictors for exacerbations. Although not conclusive, this study suggested that there were two important variables that may be related to exacerbation risk: smoking status, as already widely acknowledged,^31^ and a sense of not having control of the disease. Both these variables could be measured during the diagnostic process as a potential indicator of patients at high risk. Such patients could be given additional help with personal communication, medicines adherence and symptom control. Given the large number of patients with COPD, it would be highly relevant to improve risk assessment so as to avert or minimise exacerbations. Very poor QoL is associated with exacerbation frequency and loss of control.

Among patients who completed SLS COPD, although reported levels of COPD control and overall QoL were similar among randomised treatment groups, treatment initiation with FF/VI was associated with perceived improvements in control and improved overall QoL over the course of the study compared with continuing usual care. Suggestions of potential relationships between smoking status and feelings of control and exacerbation risk were exploratory in nature and should be confirmed prospectively in future studies.

## Methods

The SLS COPD study design and methodology for SLS COPD follow-up interviews have been described previously.^18,19^ In brief, SLS COPD was a 12-month, open-label, randomised, parallel-group, Phase III clinical trial conducted in a primary care setting in Salford in the United Kingdom; participants were recruited between March 2012–October 2014. Patients aged ≥40 years with COPD who experienced ≥1 exacerbation in the previous 3 years and who were receiving regular maintenance inhaler therapy were randomised to either initiate treatment with a once-daily, inhaled combination of FF/VI 100/25 μg or to continue with usual care as determined by their general practitioner. Randomisation (1:1) was stratified according to baseline maintenance therapy (receipt of LABA, long-acting muscarinic antagonist [LAMA] or LABA/LAMA; ICS, ICS/LABA or ICS/LAMA; or ICS/LABA/LAMA) and presence or absence of a COPD exacerbation in the previous 12 months. Patients randomised to FF/VI who had been previously treated with two long-acting bronchodilators and an inhaled glucocorticoid were allowed to continue taking a long-acting muscarinic antagonist in addition to FF/VI.

SLS COPD follow-up interviews were conducted with a subset of patients completing SLS COPD within 2 weeks of exit from the study. The methods were performed in accordance with relevant guidelines and regulations and approved by the Proportionate Review Sub-Committee of the Health Research Authority (formerly the National Research Ethics Service) East Midlands’ Research Ethics Committee. All patients provided written, informed consent.

### Study objectives

The follow-up study aimed to complement the findings of SLS COPD study by providing additional descriptive information of the treatment outcomes as perceived by patients. In particular, the follow-up study focused on patient-centred outcomes beyond those captured by standardised instruments administered in SLS COPD, such as symptom experience, impact on daily life and overall QoL. The primary objective of the SLS COPD follow-up study was to determine the background and lifestyle characteristics of a subset of patients exiting SLS COPD and to describe the experiences of all SLS COPD follow-up interview participants together. Those results are reported elsewhere.^19^ The secondary objectives, reported here, were to investigate how these patient characteristics and patient-centred outcomes relate to the randomised treatment group, as well as to exacerbation rates in SLS COPD. We firstly assessed key patient-centred variables according to the treatment group that patients were randomised to in SLS COPD, i.e., treatment initiated with FF/VI 100/25 μg versus continuing usual care. Secondly, we evaluated the relationship between key patient-centred variables and the number of moderate or severe COPD exacerbations patients experienced during SLS COPD: no exacerbations, one exacerbation, and two or more exacerbations. Moderate or severe exacerbations were defined during SLS COPD as any worsening of respiratory symptoms that led to treatment with antibiotic agents or systemic glucocorticoids (or both), to hospital admission, or to scheduled or unscheduled hospital visits.

### Questionnaires

Outcomes assessed in the secondary analysis were similar to those assessed for the primary analysis of the follow-up interviews.^19^ Data were collected via structured, closed-ended questions on the following topics: background and lifestyle information; COPD symptoms; the impact of COPD on daily life; environmental and temporal COPD trigger factors; self-management of COPD and disease awareness; perceived control over COPD; experience and management of COPD exacerbations; and overall and change in QoL since the start of SLS COPD. Interviews were conducted by trained interviewers from the SLS follow-up interview team by telephone or face-to-face at the patient’s home or general practitioner’s office if the patient preferred. All interviews were conducted within 1–2 weeks of the patients completing their SLS COPD end-of-study visit and patient identifiable information was removed from the study data prior to analysis.

Background and lifestyle information were gathered from all participants, including sociodemographics, disease perceptions, general health, smoking, use of aids and adaptations, alcohol consumption and exercise. Patients were asked which COPD symptoms, and the symptoms associated with having COPD, they experienced during SLS COPD, which symptom they thought had improved the most and which symptom they thought had worsened the most since the start of SLS COPD.

The impact of COPD on daily life was explored across five domains (functioning, activity limitations, relationships, psychological impact and independence). Participants indicated the degree of difficulty or extent to which COPD interfered with each life area (not at all/a little/quite a lot/very much/unable to do). Patients were also asked how the impact they perceived COPD to have on that domain affected their QoL (not at all/a little/quite a lot/very much), and whether the impact on their QoL had changed over the course of SLS COPD study (improved a lot/improved a little/no change/became a little worse/became a lot worse). Patients were also asked to rate their overall QoL using a 10-point response scale (1 = worst possible QoL, 10 = best possible QoL) and were asked how their overall QoL had changed since the start of SLS COPD (a lot better/a little better/no change/a little worse/a lot worse). The use of a global scale was intended to provide an efficient assessment of overall QoL from the patient perspective. This ten-point scale was chosen in order to capture information separate from yet complementary to the original SLS COPD endpoints.

Environmental and temporal trigger factors were investigated through four questions. Patients were asked if they avoided certain environments (cold/damp or windy weather, smoky/dusty or overly warm/airless places) and whether their avoidance of these had changed since the start of the study. Patients were also asked whether their symptoms worsened if they became anxious or upset, and if their symptoms were worse at particular times of day.

Self-management and disease awareness explored how patients manage their COPD in their day-to-day lives. In addition, patients’ perceived control over COPD and change in perceived control was measured. Experience and management of exacerbations consisted of questions exploring the experience of exacerbations, the patients’ awareness of exacerbations and how they managed their last exacerbation. An exacerbation was defined in the interview schedules as ‘an episode when your symptoms become much worse, and you need to change your treatment, or you may need to seek medical help’.

In addition, all participants completed the CASIS and Adherence Starts with Knowledge-12 questionnaires, to assess sleep impairment and barriers to treatment adherence, respectively.^32,33^

### Statistical analyses

A follow-up sample size of 400 patients from a total population of 2000 patients in SLS COPD was determined to provide a 4.4% margin of error at a confidence level of 95%, using Cochran’s (1977) formulas for sample size. Thus, the follow-up sample is of sufficient size to provide 95% confidence that results obtained for a given outcome in the overall SLS COPD population will be ±4.4% those obtained for the follow-up sample.

The follow-up interview data were analysed descriptively using SAS 9.4 (SAS Institute INC.; Cary, North Carolina, United States). The focus of the analysis was to describe the characteristics and experiences of patients exiting SLS COPD, rather than to test specific study hypotheses, and no inferential statistical tests were performed. Categorical variables (e.g., gender, smoking status) were presented as frequency and percentage distributions. Ordinal variables (e.g., Likert-type responses to questions about impact of COPD on daily life) were presented as frequencies and percentages and/or means with SD as appropriate for the individual variable (when data are normally distributed). Continuous variables (e.g., overall QoL and CASIS scores) were presented as means and SD, medians and first and third quartiles and score ranges. Mean values were not produced as part of the overall SLS COPD analysis plan but were produced as post-hoc analyses. Subgroup analyses were undertaken, but no statistical tests of group differences were conducted.

### Reporting Summary

Further information on experimental design is available in the Nature Research Reporting Summary linked to this article.

## Supplementary information

Supplementary Table 1

Reporting Summary

## Data Availability

Access to the data sets supporting the conclusions of this manuscript may be obtained via https://www.clinicalstudydatarequest.com/.

## Supplementary information

**Supplementary information** accompanies the paper on the *npj Primary Care Respiratory Medicine* website (10.1038/s41533-019-0123-0).
