# Supplementary material for: Follow-up interviews from The Salford Lung Study (COPD) and analyses per treatment and exacerbations
Source: NPJ Prim Care Respir Med. 2019 May 9;29:20. doi: 10.1038/s41533-019-0123-0 (PMC6509249; doi:10.1038/s41533-019-0123-0)
Supplement: Supplementary file 1 — Supplementary Table 1 [file 41533_2019_123_MOESM1_ESM.pdf]

Supplementary Materials

Supplementary Table 1. Participant demographic characteristics according to the number of COPD exacerbations experienced during SLS COPD

Demographic characteristics	*n* = 400	Number of exacerbations in SLS COPD study		
	n	0 (*n* = 125)	1 (*n* = 93)	≥2 (*n* = 182)
Mean age, years (SD)	400	66.8 (9.7)	65.5 (9.3)	66.2 (9.0)
Gender**,** *n* (column %)				
Male	213	78 (62.4)	56 (60.2)	79 (43.4)
Female	187	47 (37.6)	37 (39.8)	103 (56.6)
Relationship status, *n* (column %)				
Cohabiting (married/living as married/civil partnership)	225	67 (53.6)	60 (64.5)	98 (53.8)
Single or divorced/separated or widowed/surviving partner	173	57 (45.6)	32 (34.4)	84 (46.2)
Other	2	1 (0.8)	1 (1.1)	0 (0.0)
Employment status, *n* (column %)				
Working full time	27	10 (8.0)	7 (7.5)	10 (5.5)
Working part time	13	4 (3.2)	3 (3.2)	6 (3.3)
Voluntary or charity work	5	2 (1.6)	1 (1.1)	2 (1.1)
Homemaker	3	0 (0.0)	0 (0.0)	3 (1.6)
Long-term sick leave	20	8 (6.4)	2 (2.2)	10 (5.5)
Retired	268	85 (68.0)	62 (66.7)	121 (66.5)
Unemployed	46	11 (8.8)	11 (11.8)	24 (13.2)
Other	18	5 (4.0)	7 (7.5)	6 (3.3)
Change in employment status since start of SLS COPD, *n* (column %)				
No change	374	115 (92.0)	86 (92.4)	173 (95.1)
Change				
Stopped working altogether	13	5 (4.0)	3 (3.2)	5 (2.3)
Long-term sick leave	1	0 (0.0)	1 (1.1)	0 (0.0)
Decreased hours	2	0 (0.0)	1 (1.1)	1 (0.5)
Increased hours	3	3 (2.4)	0 (0.0)	0 (0.0)
Changed job or work duties	2	0 (0.0)	0 (0.0)	2 (1.1)
Changed due to another reason	13	6 (4.8)	4 (4.3)	2 (1.1)
Missing	5	2 (1.6)	2 (2.2)	1 (0.5)

N/A not available, *SD* standard deviation, *SLS COPD* Salford Lung Study in patients with chronic obstructive pulmonary disease

Supplementary Table 2: Participant health characteristics and lifestyle information reported in SLS COPD follow-up interviews, according to the number of COPD exacerbations experienced during SLS COPD

	n = 400	Number of exacerbations in SLS COPD study		
		0	1	≥2
	n	(*n* = 125)	(*n* = 93)	(*n* = 182)
Health characteristics				
Long-term illness or health problems in addition to COPD, *n* (column %)[a]				
Condition that limits physical activities or mobility	175	57 (45.6)	40 (43.0)	78 (42.9)
Psychological or emotional condition	21	12 (9.6)	4 (4.3)	5 (2.7)
Other	116	34 (27.2)	28 (30.1)	54 (29.7)
None	148	44 (35.2)	32 (34.4)	72 (39.6)
Missing	4	1 (0.8)	0 (0.0)	3 (1.6)
Healthcare practitioner generally seen for COPD, *n* (column %)[a]				
GP	360	108 (86.4)	84 (90.3)	168 (92.3)
Practice nurse	342	111 (88.8)	78 (83.9)	153 (84.1)
Hospital specialist	35	7 (5.6)	5 (5.4)	23 (12.6)
Respiratory nurse	60	17 (13.6)	10 (10.8)	33 (18.1)
Physiotherapist	5	0 (0.0)	3 (3.2)	2 (1.1)
Missing	1	0 (0.0)	0 (0.0)	1 (0.5)
Lifestyle information				
Smoking status, *n* (column %)				
Not smoking[b]	240	81 (64.8)	55 (59.1)	104 (57.1)
Smoking	148	41 (32.8)	33 (35.5)	74 (40.7)
Missing	12	3 (2.4)	5 (5.4)	4 (2.2)
Exercise undertaken, *n* (column %)				
Any exercise	311	94 (75.2)	77 (82.8)	140 (76.9)
Breathing exercise	141	35 (28.0)	33 (35.5)	73 (40.1)
Flexibility exercise	171	53 (42.4)	37 (39.8)	81 (44.5)
Resistance exercise	46	13 (10.4)	11 (11.8)	22 (12.1)
Gentle aerobic exercise	222	73 (58.4)	58 (62.4)	91 (50.0)
High-intensity aerobic exercise	21	6 (4.8)	4 (4.3)	11 (6.0)

[a]Participants could select more than one response. [b]Includes participants who never smoked and participants who had given up smoking

Supplementary Table 3: COPD symptom variability, awareness and management of COPD exacerbations, according to number of COPD exacerbations experienced during SLS COPD

	All patients *n* = 400	SLS COPD Exacerbation Rate		
		0 (*n* = 125)	1 (*n* = 93)	≥2 (*n* = 182)
COPD symptom variability, *n*	*n* = 400	*n* = 125	*n* = 93	*n* = 182
COPD symptoms get worse if anxious or upset, *n* (% of column)				
Yes, a lot	120	24 (19.2)	32 (34.4)	64 (35.2)
Yes, a little	110	34 (27.2)	24 (25.8)	52 (28.6)
No, not at all	138	55 (44.0)	31 (33.3)	52 (28.6)
Not applicable	28	11 (8.8)	6 (6.5)	11 (6.0)
Missing	4	1 (0.8)	0 (0.0)	3 (1.6)
Awareness of COPD exacerbations, *n*	*n* = 266	*n* = 58	*n* = 62	*n* = 146
Severity of last exacerbation, *n* (% of column)				
Mild	21	6 (10.3)	5 (8.1)	10 (6.8)
Moderate	74	19 (32.8)	19 (30.6)	36 (24.7)
Severe	127	23 (39.7)	33 (53.2)	71 (48.6)
Very severe	42	10 (17.2)	5 (8.1)	27 (18.5)
Missing	2	0 (0.0)	0 (0.0)	2 (1.4)
Aware when an exacerbation about to happen? *n* (% of column)				
Yes	169	31 (53.4)	36 (58.1)	102 (69.9)
No	97	27 (46.6)	26 (41.9)	44 (30.1)
First course of action for most recent exacerbation,[a] *n* (% of column)				
Managed symptoms at home	161	35 (60.3)	31 (50.0)	95 (65.1)
Sought immediate medical help[b]	99	23 (39.7)	30 (48.4)	46 (31.5)
Missing	6	0 (0.0)	1 (1.6)	5 (3.4)
Self-management of COPD exacerbations	*n* = 400	*n* = 125	*n* = 93	*n* = 182
Activity,[c] median (IQR)				
Pace self	398	3.0; (2.0–4.0)[d]	3.0; (2.0–3.0)[g]	3.0; (2.0–4.0)[i]
Plan activities carefully	390	1.0;(1.0–3.0)[e]	2.0; (1.0–3.0)[h]	3.0; (1.0–3.0)[j]
Accept help for everyday tasks	395	2.0; (1.0–3.0)[f]	1.0; (1.0–3.0)	2.0; (1.0–3.0)[k]
Accept help for personal care	400	1.0; (1.0–1.0)	1.0; (1.0–1.0)	1.0; (1.0–1.0)[i]
Take regular exercise	394	2.0; (1.0–3.0)	2.0; (1.0–3.0)	2.0; (1.0–3.0)

[a]Missing, *n* = 6 (2.3%). [b]Includes only respondents whose first course of action was “sought immediate medical help” (n = 99); patients could select more than one response. [c]Level of activity assessed as: 1, not at all; 2, a little; 3, quite a lot; and 4, very much. [d]*n* = 124. [e]*n* = 123. [f]*n* = 121. [g]*n* =92. [h]*n* = 91. [i]*n* = 182. [j]*n* = 176. [k]*n* = 181
GP general practitioner; *SLS COPD* Salford Lung Study in patients with chronic obstructive pulmonary disease
